# Sulfa Use, Dihydropteroate Synthase Mutations, and *Pneumocystis jirovecii* Pneumonia

**DOI:** 10.3201/eid1010.040362

**Published:** 2004-10

**Authors:** Cheryl R. Stein, Charles Poole, Powel Kazanjian, Steven R. Meshnick

**Affiliations:** * University of North Carolina at Chapel Hill, Chapel Hill, North Carolina, USA;; † University of Michigan, Ann Arbor, Michigan, USA

**Keywords:** dihydropteroate synthase, meta-analysis, Pneumocystis, Pneumocystis carinii, trimethoprim-sulfamethoxazole combination (sulfa drugs), treatment failure, research

## Abstract

A systematic review was conducted to examine the associations in *Pneumocystis jirovecii* pneumonia (PCP) patients between dihydropteroate synthase (DHPS) mutations and sulfa or sulfone (sulfa) prophylaxis and between DHPS mutations and sulfa treatment outcome. Selection criteria included study populations composed entirely of PCP patients and mutation or treatment outcome results for all patients, regardless of exposure status. Based on 13 studies, the risk of developing DHPS mutations is higher for PCP patients receiving sulfa prophylaxis than for PCP patients not receiving sulfa prophylaxis (p < 0.001). Results are too heterogeneous (p < 0.001) to warrant a single summary effect estimate. Estimated effects are weaker after 1996 and stronger in studies that included multiple isolates per patient. Five studies examined treatment outcome. The effect of DHPS mutations on treatment outcome has not been well studied, and the few studies that have been conducted are inconsistent even as to the presence or absence of an association.

## Abstract 2

Meta-analysis shows increased risk for DHPS mutations in patients exposed to sulfa prophylaxis for PCP, but clinical relevance of mutations is not known.

*Pneumocystis jirovecii* causes pneumonia in immunocompromised persons, especially those with AIDS, worldwide (*1*). In industrialized countries, while the incidence of *Pneumocystis jirovecii* pneumonia (PCP) has declined substantially since highly active antiretroviral therapy (HAART) was introduced in 1996 (*2*), PCP remains the leading serious opportunistic infection (*3**–**5*). Not all patients treated with HAART have CD4-cell count boosts above the range at which PCP occurs (*6**–**9*). In developing countries, where only 7% of HIV/AIDS patients who need therapy have access to HAART (*10*), the incidence of PCP is increasing.

Prophylaxis against PCP has been standard practice in industrialized countries for >20 years. Trimethoprim-sulfamethoxazole (TMP-SMX) is the first-line drug choice for both prophylaxis and therapy. TMP-SMX acts in animals as sulfa monotherapy against the enzyme dihydropteroate synthase (DHPS) (*11**,**12*). Dapsone, a sulfone drug also targeting DHPS, is frequently used as a second-line agent for prophylaxis and treatment of PCP.

Failure of sulfa or sulfone (sulfa) prophylaxis against PCP has been reported in up to one fourth of patients (*13**,**14*). To assess the role of drug resistance in these failures, investigators examined whether DHPS mutations are more frequent among patients with or without prior exposure to sulfa agents, and whether infections in patients with or without DHPS mutations are more likely to be unresponsive to a sulfa drug. These studies are hampered by scientists' inability to culture *P. jirovecii*, which prevents direct confirmation of resistance through standard drug-susceptibility testing. Instead, researchers use polymerase chain reaction to detect *P. jirovecii* DHPS mutations that cause sulfa resistance in other microorganisms. DHPS mutations in *P. jirovecii* may also increase the incidence of treatment failure. A systematic review can determine whether available studies give overall evidence of an association, assess the possibility of publication bias, examine results across studies for consistency, and investigate study and patient characteristics for possible influence on study results.

## Methods

### Literature Search

MEDLINE (National Library of Medicine, Bethesda, MD) was searched with the keywords "*Pneumocystis*," "*Pneumocystis carinii*," and "drug resistance" (last searched January 2004). ISI Web of Science (Institute for Scientific Information, Philadelphia, PA) was searched with the keywords "pneumocystis pneumonia," "resistance," and "genes" (last searched January 2004). The bibliographies of relevant articles were surveyed for additional studies. One author (S.R.M.) contacted 42 scientists through an informal PCP email forum to request unpublished results and conference abstracts on associations between sulfa prophylaxis and *Pneumocystis* mutations and *Pneumocystis* mutations and sulfa treatment outcome.

### Information Extraction

Inclusion requirements were the following: study populations composed entirely of PCP patients; mutation results for all patients, regardless of sulfa prophylaxis exposure; and treatment outcome results for all patients, regardless of mutation status. Studies reporting the outcome (mutation status or treatment failure) only for exposed patients (on prophylaxis or with mutations) were not included because these studies would have biased the analyses by not providing information on unexposed populations for comparison. When more than one article reported on the same study population, only the more comprehensive article was included. From every eligible report, one author extracted information on publication year, study location(s), study start and end dates for calculating data collection calendar midpoint, study size, proportion of HIV-positive patients, number of isolates per patient, timing of prophylaxis in relation to PCP, treatment outcome definition, number and type of DHPS mutations in patients receiving or not receiving sulfa prophylaxis, and sulfa treatment outcome among patients with and without DHPS mutations. Multiple isolates from the same patient were included as independent counts of PCP.

### Statistical Analysis

STATA Version 8.2 (Stata Corp., College Station, TX) was used to analyze estimates of the effect of prophylaxis on mutation occurrence and estimates of the effect of mutation on treatment outcome. Both analyses used the risk difference (RD) as the effect measure. Qualitatively similar results were obtained by using the risk ratio and incidence odds ratio (*15*). The number of patients needed to treat (NNT) to increase or decrease the number of outcomes by one may be computed as NNT = RD^–1^ (*16*). The 95% confidence limit difference (CLD), computed as the difference between the upper and lower limits of the 95% confidence interval (CI), was used to gauge the precision of the study-specific RD estimates, with smaller values denoting more precise estimates (*17*). We obtained p values for overall association from the meta-analysis of RD estimates by means of the Mantel-Haenszel test statistic. The potential for publication bias was assessed by visually examining funnel plots of RD estimates and by using standard tests of funnel plot asymmetry (*18**,**19*). Homogeneity test statistics and their associated p values were computed to assess the consistency of estimated RDs across studies. Random-effects meta-regression and stratified analyses were used to estimate associations between RD estimates and characteristics of studies and patients. The precision-weighted meta-regression models incorporated random effects by using a restricted maximum likelihood method to estimate the among-study variance (*20*).

## Results

Thirteen eligible studies were identified for the analysis of the effect of prophylaxis on mutation (*21**–**33*) and five for the analysis of mutation effect on treatment outcome (Table 1) (*25**–**27**,**34**,**35*). Three studies were included in both analyses (*25**–**27*).

**Table 1:** Study characteristics and effect estimates

Study	Location/data collection calendar midpoint^a^	N	Multiple isolates per patient	Defined prophylaxis timing^b^	Defined treatment outcome^c,d^	Proportion HIV+	RD (95% CI) (95% CLD)^d^
Prophylaxis effect on mutation							
Kazanjian (1998) (*21*)	USA/1994	27	No	Yes	NA	0.74	0.61 (0.25, 0.97) (0.72)
Helweg-Larsen (*22*)	Denmark/1994	152	Yes	Yes	NA	1.00	0.51 (0.33, 0.70) (0.37)
Ma (1999) (*23*)	USA/1992	37	Yes	Yes	NA	0.70	0.69 (0.43, 0.94) (0.51)
Huang (*24*)	USA/1998	111	No	Yes	NA	1.00	0.33 (0.15, 0.51) (0.36)
Kazanjian (2000) (*25*)	USA/1995	97	No	Yes	NA	1.00	0.52 (0.35, 0.70) (0.35)
Visconti (*26*)	Italy/1995	20	Yes	No	NA	1.00	0.60 (0.20, 1.00) (0.80)
Ma (2002) (*27*)	Italy/1998	107	No	Yes	NA	1.00	0.15 (0.01, 0.30) (0.29)
Costa (*28*)	Portugal/1998	89	No	Yes	NA	0.93	0.10 (–0.15, 0.35) (0.50)
Crothers (*29*)	USA/2000	236	Yes	Yes	NA	1.00	0.16 (0.06. 0.25) (0.19)
Latouche (*30*)	France/2000	92	No	Yes	NA	0.90	–0.03 (–0.22, 0.16) (0.38)
Miller (*31*)	England/1993	25	No	Yes	NA	1.00	0.31 (–0.08, 0.69) (0.77)
Nahimana (*32*)	France/1995	158	No	Yes	NA	0.76	0.50 (0.31, 0.69) (0.38)
Zingale (*33*)	Italy/1999	64	Yes	Yes	NA	1.00	0.61 (0.42, 0.80) (0.38)
Mutation effect on treatment outcome							
Kazanjian (2000) (*25*)	USA/1995	97	No	NA	Yes	1.00	0.22 (0.01, 0.43) (0.42)
Takahashi (*34*)	Japan/1997	24	No	NA	Yes	0.67	0.89 (0.59, 1.19) (0.60)
Ma (2002) (*27*)	Italy/1998	107	No	NA	NA	1.00	–0.01 (–0.22, 0.20) (0.42)
Navin (*35*)	USA/1997	136	No	NA	Yes	1.00	–0.21 (0.39, –0.03) (0.36)
Visconti (*26*)	Italy/1995	20	Yes	NA	No	1.00	–0.21 (–0.82, 0.40) (1.22)

^a^Data collection calendar midpoint, the midpoint in calendar time of data collection. ^b^Defined prophylaxis timing, whether the study stated the timing of prophylaxis in relation to the episode of *Pneumocystis jirovecii* pneumonia. ^c^Defined treatment outcome, whether the study stated how it defined treatment outcome. ^d^RD, risk difference; CI, confidence interval; CLD, confidence limit difference; NA, not applicable.

### Prophylaxis Effect on Mutation

In this analysis, the estimated RD from each study is the risk of developing a DHPS mutation among PCP patients exposed to sulfa prophylaxis minus the risk among PCP patients not exposed to sulfa prophylaxis. The RD meta-analysis produced strong evidence of a positive association (p < 0.001). Twelve of the 13 studies reported results suggesting that prophylaxis increases the risk for DHPS mutations, and 95% CI of 10 of the 13 excluded the null value (Table 1, Figure). The 12 positive RD estimates ranged from a 10% increase in risk (*28*) to a 69% increase (*23*). The least precise estimate came from a study with only 20 isolates (*26*), and the most precise estimate from a study with 236 (*29*). Visual inspection of the funnel plot, Begg and Mazumdar's test (p = 0.5), and the test of Egger et al. (p = 0.1) all gave no appreciable evidence of asymmetry. The study-specific results were highly heterogeneous (p < 0.001), however. As shown in the Figure, the 95% CI for three estimates (*27**,**29**,**30*) did not overlap the CI for five other estimates (*22**,**23**,**25**,**32**,**33*). No single summary estimate can adequately describe results as disparate as these (*36*).

**Figure:**
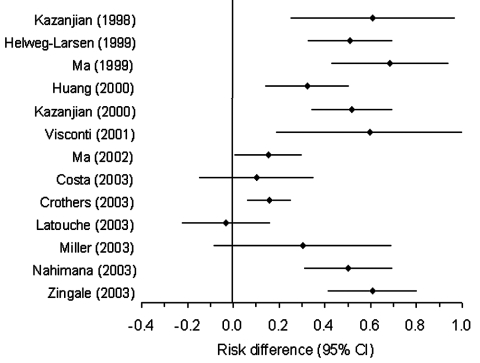
Forest plot, prophylaxis effect on mutation. CI, confidence intervals.

Of the examined characteristics, data collection calendar midpoint and multiple isolates both had strong associations with the study results (Table 2). Higher estimated RDs were produced by studies in which at least half of the data was collected before 1996 (*21**–**23**,**25**,**26**,**31**,**32*) and from studies including multiple isolates per patients (*22**,**23**,**26**,**29**,**33*). Three studies had a data collection calendar midpoint before 1996 and used multiple isolates per patient (*22**,**23**,**26*). The magnitude of the combined influence of these two characteristics on the estimate (difference of RD = 0.10, 95% CLD 0.22) was less than either of the individual characteristics examined singly. Only two studies with a midpoint of 1996 or later included multiple isolates from the same patient (*27**,**28*).

**Table 2:** Stratified and random-effects meta-regression analysis of study characteristics

Study characteristic^a^	Characteristic level	No. of studies	RD (95% CLD)^b^	Homogeneity test p value	Difference of RDs (95% CLD)^b^
Prophylaxis effect on mutation					
Data collection calendar midpoint	1996 or later	6	0.22 (0.31)	< 0.001	–0.32 (0.39)
	Before 1996	7	0.53 (0.18)	0.8	0
	4-y change	13	NA	NA	–0.23 (0.30)
Prophylaxis use by					
Specific mutations	Yes	4	0.54 (0.24)	0.6	0.20 (0.55)
	No	9	0.32 (0.29)	< 0.001	0
Multiple isolates per patient	Yes	5	0.50 (0.50)	< 0.001	0.19 (0.52)
	No	8	0.30 (0.32)	< 0.001	0
Location	USA	5	0.44 (0.43)	< 0.001	0.10 (0.55)
	Outside USA	8	0.34 (0.36)	< 0.001	0
Defined treatment outcome	Yes	5	0.32 (0.44)	< 0.001	–0.08 (0.54)
	No	8	0.41 (0.35)	< 0.001	0
Multicenter	Yes	5	0.41 (0.31)	0.0	0.05 (0.55)
	No	8	0.36 (0.36)	< 0.001	0
Proportion HIV+	1.00	8	0.38 (0.30)	< 0.001	0.03 (0.56)
	< 1.00	5	0.37 (0.58)	< 0.001	0

^a^Data collection calendar midpoint, the midpoint in calendar time of data collection; defined treatment outcome, whether the study stated how it defined treatment outcome. ^b^RD, risk difference; CLD, confidence limit difference; NA, not applicable.

The four studies that detailed prophylactic drug use for each specific mutation had a high homogeneity p value (p = 0.6) and a higher estimated RD (Table 2) (*22**,**26**,**31**,**33*). One study did not provide information on the timing of sulfa prophylaxis in relation to the PCP episode (*26*). With this study removed so its influence on the meta-analysis could be evaluated, the homogeneity p value remained low for the other 12 studies (p < 0.001). The remaining characteristics were weakly associated with study results.

### Mutation Effect on Treatment Outcome

In this analysis, the estimated RD from each study is the risk of failing sulfa treatment for PCP among patients with DHPS mutations minus the risk among patients without DHPS mutations. Five studies provided such a result. One of these studies had a mixed HIV-positive and HIV-negative patient population (*34*), and another did not describe the criteria for determining treatment outcome (*26*). Three of the studies included in the analysis of prophylaxis effect on mutation (*22**,**30**,**32*) mentioned examining treatment outcome but did not provide usable treatment outcome data for the full study population.

Assessing publication bias was impractical with only five published studies. Two of the five suggested that patients infected with mutant *P. jirovecii* were unexpectedly more likely to be responsive to treatment for PCP (Table 1) (*26**,**35*). One study showed that mutations had virtually no effect (*27*). The remaining two studies were on the opposite side of the null hypothesis (Table 1) (*25**,**34*). The pronounced evidence of heterogeneity (p < 0.001) was easily discerned by examining CI nonoverlap, since the 95% CI for the study with the highest estimate for increased risk (*34*) did not overlap any of the other four CIs.

## Discussion

PCP patients receiving sulfa prophylaxis are at increased risk for DHPS mutations compared with PCP patients not receiving sulfa. The strength of the association varies greatly across studies.

Not all studies adhered to a uniform definition of substantive sulfa exposure. Only some defined a minimum duration of prophylaxis use, often in conjunction with the timing of the PCP episode, for a patient to be counted as receiving prophylaxis. Some studies were more comprehensive in documenting prophylactic drug use by pulling pharmacy records to verify that prophylactic medications were dispensed or patient questionnaires to confirm that the drug was taken. Moreover, the association between prophylactic drug use and mutation was stronger for the studies that included multiple isolates than for those that did not. This difference suggests the possibility that exposure to multiple courses of sulfa prophylaxis increases the chance of developing DHPS mutations. The weakened association evident since 1996 may reflect a higher overall prevalence of mutation with a higher prevalence among those unexposed to prophylaxis, or it may reflect that fewer HIV-infected patients take prophylactic drugs because of HAART. Each of these factors may bear on the strength of the association between sulfa prophylaxis and DHPS mutations. Variations in unreported aspects of study design or patient characteristics may account for the remaining inconsistency in estimated effect size.

One of the 13 studies reported an inverse association between prophylaxis and mutations (*30*). Unlike the other studies, this study categorized prophylaxis use as regular, irregular, none, and unknown. We categorized sulfa exposure as regular or irregular use. Had we counted only regular prophylactic drug use as sulfa exposure, the association in this study would have been positive, albeit very imprecise (RD = 0.17, 95% CLD = 1.32). Additionally, the isolates in this study were collected more recently than in other studies, with all specimens collected after 1998.

This systematic review was unable to resolve the conflicting results regarding the magnitude of the effect of DHPS mutations on treatment outcome. Only five studies were eligible for inclusion in this analysis. Although the small number of studies precluded a statistical investigation of possible explanations for the inconsistent findings, variation in definitions of treatment outcome may be partially responsible. The two studies with positive associations used clinical improvement after therapy to determine treatment outcome (*25**,**34*), whereas the two studies with negative associations defined treatment outcome as survival after the episode (*26**,**35*). The treatment outcome definition for the study showing minimal effect used both survival and clinical recovery without relapse (*27*). HIV status may also have swayed the results. The sole study to include HIV-uninfected patients noted the strongest association between mutation and treatment outcome (*34*).

This systematic review has both strengths and limitations. It included information on all relevant studies for which results have been reported, examined how different study characteristics influenced the magnitude of effect estimates, and provided information that may be useful when designing future studies of a similar nature. Its principal weakness was that the small number of available studies, especially for treatment outcome, made the results from stratified analysis and meta-regression less precise than would be desirable.

We conclude that exposure to sulfa prophylaxis for PCP increases the risk for DHPS mutations. This finding is evident even with the heterogeneity of the individual study results. Although whether these mutations are clinically relevant is unclear, they are likely to develop in patients who have received sulfa prophylaxis for PCP for extended periods. This review did not clarify the effect of these mutations on treatment outcome. Further studies are needed to examine the association between DHPS mutations and treatment outcome in patients with PCP. Until these studies are performed, the optimal treatment for patients with PCP, who have had substantive exposure to sulfa prophylaxis and who are therefore likely to have DHPS mutations, remains speculative.
